# Real-time RT–PCR correlates with immunocytochemistry for the detection of disseminated epithelial cells in bone marrow aspirates of patients with breast cancer

**DOI:** 10.1038/sj.bjc.6602189

**Published:** 2004-10-26

**Authors:** I H Benoy, H Elst, I Van der Auwera, S Van Laere, P van Dam, E Van Marck, S Scharpé, P B Vermeulen, L Y Dirix

**Affiliations:** 1 Translational Cancer Research Group Antwerp (Lab Pathology University of Antwerp/University Hospital Antwerp, 2650 Edegem; Oncology Centre, General Hospital Sint-Augustinus, 2610 Wilrijk, Belgium), Belgium; 2 Medical Biochemistry, University of Antwerp, 2610 Wilrijk, Belgium

**Keywords:** RT–PCR, breast cancer, bone marrow, circulating tumour cells, cytokeratin-19, mammaglobin

## Abstract

Real-time reverse transcriptase–polymerase chain reaction (RT–PCR) is a technique with the potential of improving the quantification of disseminated epithelial cells (DEC) in haematological tissues due to its exquisite sensitivity. This sensitivity may lead to false positivity. Immunocytochemistry (ICC) is regarded as the standard methodology to diagnose DEC. In this study, detection with ICC was compared with quantitative real-time RT–PCR for CK-19 and mammaglobin (hMAM) mRNA in bone marrow (BM) of patients with metastatic breast cancer (MBC). Bone marrow was aspirated from 14 control patients and from 29 patients with MBC. Mononuclear cells (MNC) were isolated. Immunostaining was carried out with the Epimet kit. Quantitative PCR was performed on the ABI Prism 7700. The CK-19 and hMAM mRNA quantities were normalised against *β*-Actin and calculated relative to a calibrator sample (relative gene expression). All controls were negative by ICC and for hMAM expression measured by RT–PCR, whereas the median RGE value for CK-19 was 0.57. For the MBC patients, the median RGE for hMAM was 0 and 10 out of 25 (40%) tested positive. Median RGE for CK-19 was 2.9 and 20 out of 25 (80%) tested positive. With ICC, the median value was 1 stained cell per sample, and 15 out of 24 (62%) samples were positive. A correlation was observed between CK-19 and hMAM expression (*r*=0.7; *P*=0.0003), and between hMAM expression and ICC (*r*=0.6; *P*=0.003). CK-19 expression and ICC (*r*=0.9; *P*<0.0001) showed the strongest correlation. Reverse transcriptase–polymerase chain reaction for CK-19 resulted in a higher number of positive BM samples of patients with MBC than ICC. Since an excellent correlation is observed between ICC and RT–PCR, and RT–PCR is probably more sensitive with the advantage of being less observer dependent and thus also more easy to automate, we consider our quantitative real-time RT–PCR method as validated for the detection of DEC in the bone marrow of breast cancer patients.

In the Western world, nearly one out of nine women develop breast cancer. The majority of these patients do not have evidence of metastatic disease using conventional diagnostic techniques. However, almost 30% of patients with stage I or stage II disease will die of metastasis, probably implying that dissemination, although undetectable, had already occurred at the moment of diagnosis.

Detection of disseminated epithelial cells (DEC) in the blood–bone marrow (BM) compartment is still in the experimental phase. Molecular diagnostic techniques have, however, been integrated in the revised tumour-node-metastasis (TNM) staging system for the detection of metastatic tumour deposits in lymph nodes of patients with breast cancer (Singletary *et al*, 2002).

The standard method to detect DEC in BM is immunocytochemistry (ICC). For patients with breast cancer, several studies demonstrated that the presence of ICC-stained cytokeratin-positive cells in the bone marrow is associated with a poor prognosis (Diel *et al*, 1996; Braun *et al*, 2000; Janni *et al*, 2000; Gerber *et al*, 2001; Singletary *et al*, 2002; Wiedswang *et al*, 2003). The value of this cytological method is limited by its low sensitivity, and is highly dependent on the experience of the observer. An additional well-known problem is the false positivity of some haematological cells (Borgen *et al*, 1999).

Since the introduction of molecular-based techniques, more sensitive quantitative methods have been developed, for instance, based on PCR methodology. This approach is aimed at the amplification of specific abnormalities present in the DNA of tumour cells. Since the common solid tumours rarely have specific genetic abnormalities, the use of DNA-based methods is precluded. The most commonly used molecular method for the detection of DEC relies on the screening for tumour-associated and/or organ-specific mRNA expression in cancer cells and on the absence of these gene products in the cells of the host tissue such as BM.

The identification of an appropriate target gene is one of the most critical steps in the reverse transcriptase–polymerase chain reaction (RT–PCR) approach to quantify DEC. Cytokeratins are widely evaluated as targets for the detection of DEC, but many studies have reported on the problem of false positivity, for example, with the detection of CK-19 mRNA, which may be explained by illegitimate expression by nonepithelial cells (Chelly *et al*, 1989; Ko *et al*, 2000). Combining markers may increase both the specificity and the sensitivity of the detection of DEC. Human mammaglobin (hMAM), a member of the uteroglobin gene family, was reported to be exclusively expressed in mammary epithelium (Watson and Fleming, 1996; Watson *et al*, 1999), making it a potentially useful RT–PCR target.

A prerequisite for the prognostic/predictive evaluation of DEC in the BM quantified by RT–PCR, and the aim of this study, is the proof of a stochastic correlation of the molecular quantitative method with ICC-based quantification of DEC. Some studies (Schoenfeld *et al*, 1997; Zhong *et al*, 1999) have adopted the molecular approach, but not in a quantitative manner, having expressed the BM as being DEC-positive or negative. Slade (1999) and Smith *et al* (2000) use a competitive quantitative RT–PCR assay and compare the results with ICC. In this study, quantification of BM DEC by ICC is compared with quantitative real-time RT–PCR quantification of CK-19 and hMAM mRNA expression in a control population and in patients with metastatic breast cancer.

## PATIENTS AND METHODS

### Patient samples

After written informed consent, BM aspirates were taken from 29 patients with metastatic breast cancer and 14 patients with a nonmalignant breast lesion or a haematological malignancy (control patients).

In total, 18 ml of BM was aspirated from the posterior iliac crest under local anaesthesia into syringes containing heparin as anticoagulant.

Mononuclear cells (MNC) were isolated by density-gradient centrifugation through Ficoll-Paque (Amersham Pharmacia Biotech, Sweden) and washed twice with PBS. The samples were then divided into two aliquots, one for each methodology. After centrifugation, the cell pellets were resuspended in a guanidine-containing buffer or in PBS, for RT–PCR and ICC, respectively.

For optimalisation of the assay, BM aspirates were also analysed from 56 consecutive patients with operable breast cancer presented in our hospital.

The study protocol was approved by the ethical committees of the Faculty of Medicine, University of Antwerp, and of the General Hospital Sint-Augustinus.

### Immunocytochemistry

The MNC suspension was counted and cytocentrifuged onto glass slides at a concentration of 5 × 10^5^ cells per spot. The cytospin preparations were air-dried overnight and then stored at −80°C. Immunostaining to detect cytokeratin-positive cells was carried out with the Epimet®-kit (Micromet AG, Germany). This kit uses the monoclonal antibody A45-B/B3, a pancytokeratin marker. A total of 2 million cells per patient were screened microscopically by two independent observers. Cells were identified as DEC according to the European ISHAGE Working group for Standardisation of Tumour Cell Detection (Borgen *et al*, 1999). Results were expressed as the number of positive cells per million MNC (continuous data). A sample was considered to be positive if one or more stained cells were observed in the cytospins (categorical data).

Sensitivity was tested on an MNC suspension from a healthy volunteer spiked with 1, 2, 10, 20, 100 and 1000 MCF-7 breast cancer cells per million MNC.

### RNA isolation and cDNA synthesis

Total RNA was extracted from the MNC using the RNeasy kit (Qiagen, Germany). The amount of RNA was measured spectrophotometrically. All samples had an OD 260/280 nm ratio >1.8, indicating high purity. The RNA integrity was tested on the Agilent Bioanalyzer. Only samples with lack of degradation on electropherogram and 28S/18S ratio were analysed.

For the generation of first strand cDNA, 2 *μ*g of total RNA was reverse transcribed with the high-capacity cDNA Archive Kit (Applied Biosystems, The Netherlands) in a total volume of 100 *μ*l.

### PCR amplification

cDNA-specific CK-19 and hMAM Taqman™ primer and probe sets were developed using Primer Express® software. To avoid amplification of contaminating genomic DNA, primers and probes were placed on different exons. The forward primer of CK-19 (CCCGCGACTACAGCCACTA) is situated on exon 1, the probe (FAM-ACCATTGAGAACTCCAGGATTGTCCTGCA-TAMRA) on exon 2 and the reverse primer (CTCATGCGCAGAGCCTGTT) on exon 3. Reverse transcriptase–polymerase chain reaction using this primer set resulted in a 163 bp fragment.

For hMAM, the forward primer (ATGAAGTTGCTGATGGTCCTCAT) and the probe (FAM-CGGCCCTCTCCCAGCACTGC-TAMRA) are located on exon 1 and the reverse primer (GTCTTAGACACTTGTGGATTGATTGTCT) on exon 2. The hMAM amplicon consists of 119 bp. The nucleotide sequences of the primers and probes were checked for their specificity in the NCBI BLAST® database.

A ready to use primer and probe set predesigned by Applied Biosystems (Assay-on demand Gene Expression Product number Hs00267190_m1, SCGB2A2) was also used for the detection of hMAM expression. Commercially available primers and probes for GAPDH and *β*-Actin mRNA were used for normalisation (Applied Biosystems). These probes are labelled with a VIC dye and to avoid competition in the multiplex PCR reaction tube, the concentrations of the primers are limited.

All PCR reactions were performed on the ABI Prism 7700 Sequence Detection System (Applied Biosystems) using the fluorescent Taqman methodology. The PCR cycle at which the fluorescence arises above the background signal is called the cycle threshold (Ct).

In total, 10 *μ*l of the reverse transcription volume was used for each PCR reaction in a total volume of 50 *μ*l. Since a multiplex PCR reaction is carried out, each reaction tube contains more than one primer pair: one primer pair amplifies the target and another pair amplifies the endogenous reference. Primer and probe concentration for the target gene were optimised according to the manufacturer's procedure. For CK-19 mRNA, the concentrations were 300 nM for forward and reverse primers and 150 nM for the probe. The forward and reverse primers for hMAM mRNA detection were used in a concentration of 900 nM and the probe in a concentration of 200 nM. The primers and probe of the predeveloped assay-on-demand were used according to the manufacturer's guidelines. The thermal cycling conditions comprised 2 min at 50°C, 10 min at 95°C and 50 cycles of 15 s denaturation at 95°C and 60 s annealing at 60°C.

The CK-19 and hMAM mRNA quantities were analysed in triplicate, normalised against *β*-Actin or GAPDH as a control gene and expressed in relation to a calibrator sample. As described by Livak and Schmittgen (2001), results are expressed as relative gene expression (RGE) using the ΔΔ Ct method. The calibrator was produced from the blood of a healthy volunteer spiked with 5 MDA-MB361 cells per 10^6^ MNC. The calibrator was given a RGE value of 100.

### Cell lines

The cell lines were cultured in Dulbecco's MEM culture medium (MCF-7 and MDA-MB231) and in Leibovitz medium for MDA-MB361. The media contained 10–20% fetal bovine serum and antibiotics. MCF-7 and MDA-MB231 cells were cultured at 37°C in a 5% CO_2_ atmosphere. The medium was replaced twice a week. MDA-MB361 cells were cultured in the normal atmosphere and the medium was replaced only once a week. Culture cells were harvested according to the American Type Culture Collection (ATCC) guidelines.

For total RNA isolation, cells were pelleted and washed twice with PBS. Pellets were resuspended in RLT buffer (Qiagen), and RNA was prepared according to the Rneasy midi protocol.

### Standard curves

A standard curve was constructed with a six-fold serial dilution of cDNA obtained from an MDA-MB361 breast cancer cell line. The standard curve was composed of six points with an equivalent of 200, 100, 40 and 20, 2 and 0.2 ng MDA-MB361 total RNA. This standard curve was presented as an *XY* scatter plot, where the *X*-axis represents the log of the input amount (log pg of starting total RNA) and the *Y*-axis the corresponding Ct value. Equations were derived from the curves.

Sensitivity of CK-19 and hMAM RT–PCR was tested on limiting dilutions of a BM aspirate spiked with MDA-MB361 cells to obtain the following concentrations: 0, 0.5, 1, 2, 5, 10, 50, 100, 1000 and 10 000 cells per 10^6^ MNC.

### Statistics

Data were analysed using the statistical software package SPSS 11.0. The Mann–Whitney *U*-test was used to validate differences in continuous variables and the Fisher's exact test was used to validate differences in categorical variables. Correlation analyses were validated by the Spearman *ρ* correlation test for continuous non parametric variables and by the *κ* test for categorical variables. The McNemar test was used to compare ICC and RT–PCR on the same BM sample.

## RESULTS

### Immunocytochemistry

By screening 2 million MNC of the healthy volunteer's blood spiked with breast cancer cells, a sensitivity of 10 tumour cells per million MNC was obtained.

In none of the 14 BM aspirates from negative control patients were cytokeratin- positive cells detectable. Cytokeratin-positive cells were counted in cytospins from 24 metastatic breast cancer patients (five patients were excluded for ICC analysis because of insufficient sample collection or poor cytospin quality). The median number of positive cells in these patient samples was one cell 10^6^ MNC^−1^ (minimum 0 and maximum 122). In 62% (15 out of 24) of the patients, at least one cell was immunostained.

### CK-19 and hMAM expression in various breast cancer cell lines

CK-19 and hMAM expressions were measured in MCF-7, MDA-MB231 and MDA-MB-361 breast cancer cell lines. CK-19 mRNA could be amplified in all three cell lines with the strongest expression in the MCF-7 cell line (Ct of 15.39, *vs* 19.50 and 18.36 in MDA-MB231 and MDA-MB361, respectively). Mammaglobin mRNA expression was not detectable in the cell line MDA-MB231. A very weak expression was found in the MCF-7 cell line (Ct of 39.07). MDA-MB361 cells had a strong hMAM expression (Ct of 14.58).

### Sensitivity of quantitative RT–PCR for CK-19 and hMAM mRNA

Five MDA-MB361 cells in a total of 10 million MNC were still detectable with the CK-19 RT–PCR test. Human mammaglobin RT–PCR was positive up to two MDA-MB361 cells per million MNC.

### Normalisation to housekeeping genes

For exact quantification of gene expression, an active and endogenous reference (housekeeping gene) is used to correct for differences in the amount of total RNA added to a reaction, for compensation of different RT efficiencies and for compensation of PCR inhibitors in the sample.

We evaluated the two most frequently used housekeeping genes for normalisation of our results: GAPDH and *β*-Actin. Primers and probes for these housekeeping genes were commercially available and according to the manufacturer, these assay reagents were designed to exclude detection of genomic DNA. These assays did not detect the genomic DNA after 35 cycles of amplification.

**Figure 1:**
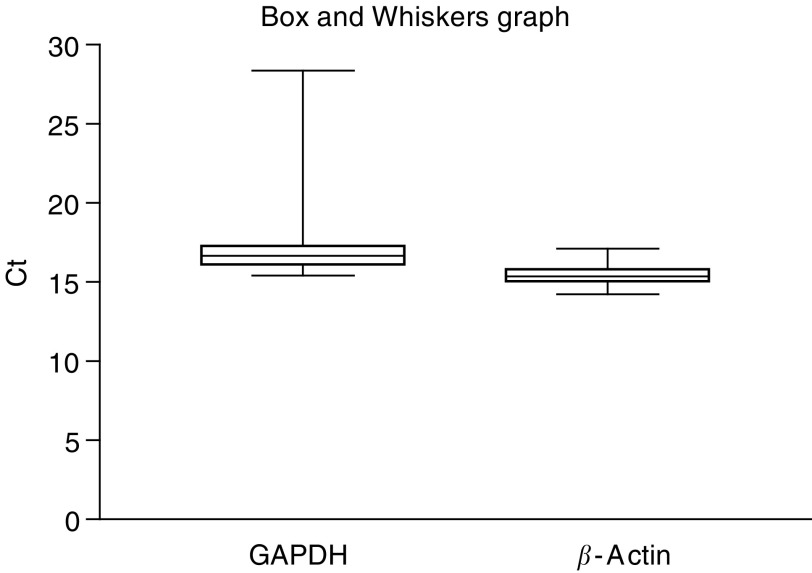
Representation of the variation in Ct value for GAPDH and *β*-Actin measured in 81 bone marrow samples.

The expression of these two markers was measured in 81 BM samples from patients with breast cancer (25 patients with metastatic disease, 56 with operable disease). Coefficients of variation were measured according to the difference in the Ct value (Figure 1

, Table 1

**Table 1:** Variation of GAPDH and *β*-Actin expression in BM samples

	**GAPDH**	***β*-Actin**
*N*	81	81
Mean Ct	17.05	15.46
s.d.	2.1	0.6
CV	12%	3.80%

*N*=number of samples analysed; Ct=cycle threshold; s.d.=standard deviation; CV=coefficient of variation; GAPDH=glyceraldehyde 3 phosphate dehydrogenase.

). Since the BM aspirates showed a wide variation in GAPDH expression (CV 12%), while *β*-Actin was expressed more stably (CV=3.8%), *β*-Actin was selected as the endogenous control.

### Validation of ΔΔ Ct method

**Figure 2:**
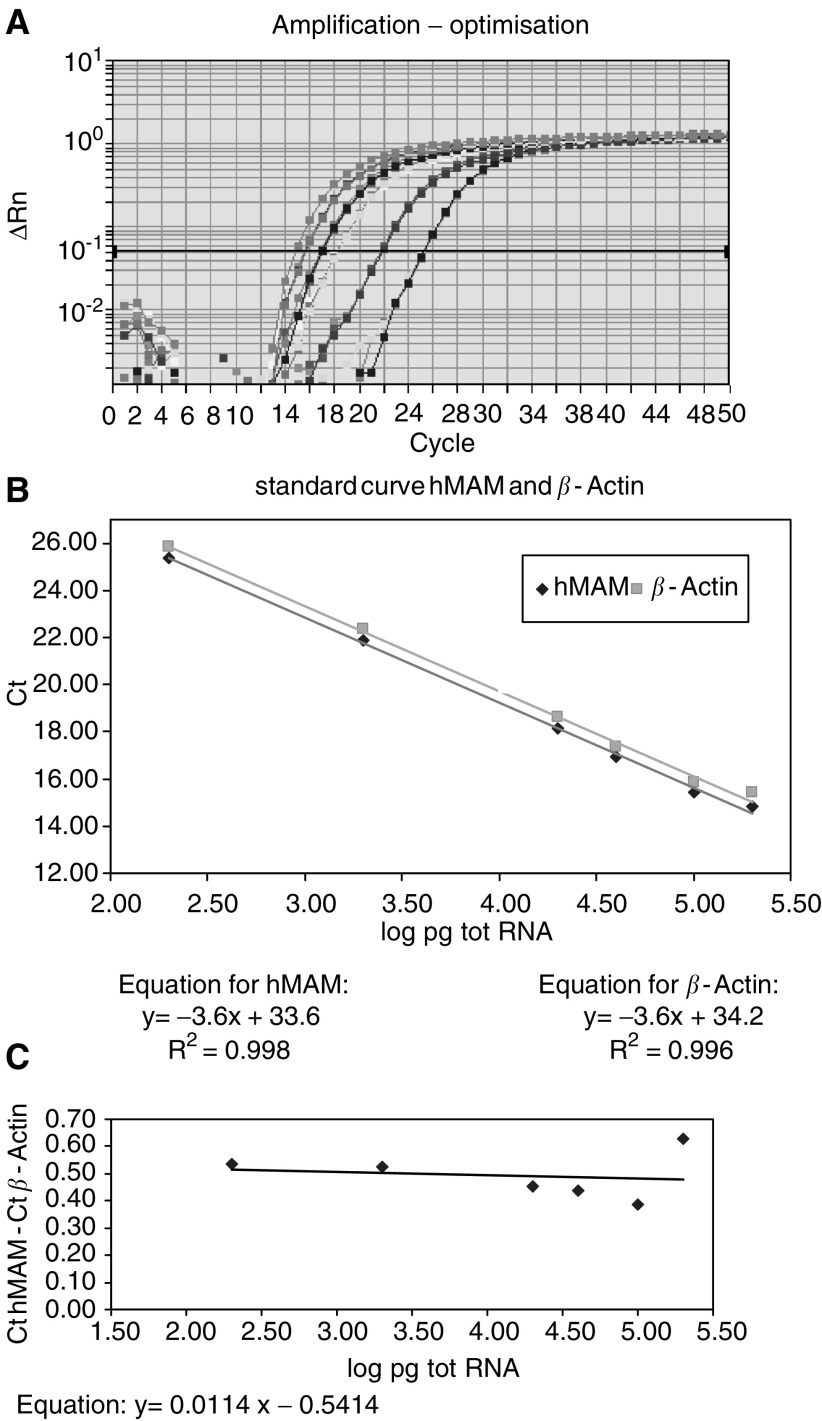
Validation of the ΔΔ Ct method for the quantification of hMAM results: (**A**) amplification plots obtained by the ABI Prism 7700 for hMAM mRNA expression; (**B**) Standard curve for hMAM and *β*-Actin constructed of six cDNA dilutions obtained from an MDA-MB361 breast cancer cell line; (**C**) Plot of the log of input of total RNA *vs* the ΔCt value (Ct hMAM - Ct *β*-Actin).

We used the ΔΔ Ct method to quantify our results. To adopt this calculation method, the amplification efficiencies of the target gene (CK-19 or hMAM) and endogenous control gene (GAPDH or *β*-Actin) have to be comparable: the slope of the plot of the log of input of total RNA *vs* the ΔCt value has to be ⩽0.1. For CK-19 *vs* GAPDH, a slope of 0.08 was found. For CK-19 *vs β*-Actin, a slope of 0.1 was found. The amplification efficiencies of hMAM and GAPDH or *β*-Actin were also equal (slopes of 0.08 and 0.01, respectively) (Figure 2

).

### Comparison of a manufacturer-designed primer and probe set and a home-made primer and probe set for the detection of hMAM mRNA

**Figure 3:**
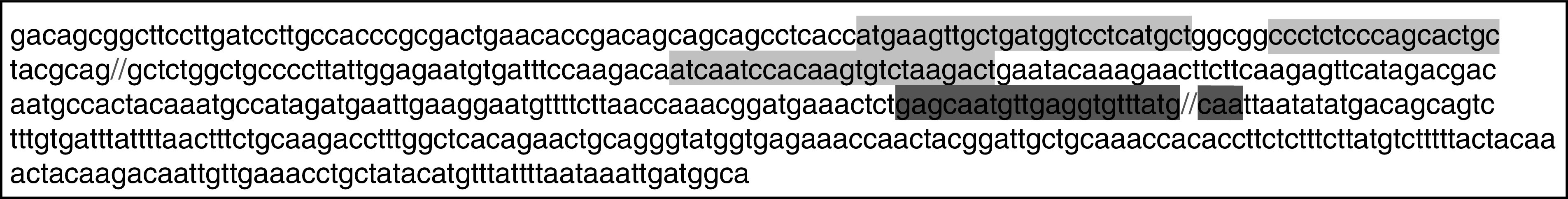
Human mammaglobin mRNA sequence: //indicates exon–intron boundary, ▪ =our own primers and probe, ▒ =neighbourhood of probe location of the assay-on-demand.

Primers and probe sequences from the Assay-on-demand (Applied Biosystems) are not revealed by the manufacturer. Only sequence context information, consisting of 25 bases randomly selected around the probe, is available. In Figure 3

, the hMAM mRNA sequence is given. The 25 bases are highlighted as are the primers and probe sequences constructed in the laboratory.

In total, 30 BM samples – six negative control patients (NC), 13 MBC patients and 11 patients with operable breast cancer (PBC) – were tested with both primer and probe sets for the quantification (RGE) of hMAM mRNA. Results are given in Table 2

**Table 2:** Human mammaglobin expression measured in 30 different bone marrow samples with two different primers and probe sets

**Patient number**	**Group**	**RGE hMAM**	**RGE hMAM AOD**
1	MBC	0.2	0.06
2	PBC	0	0
3	PBC	0.78	0.60
4	MBC	0	0
5	MBC	0	0
6	NC	0	0
7	PBC	0	0
8	MBC	262.68	86.06
9	NC	0	0
10	MBC	27.29	9.1
11	NC	0	0
12	PBC	0	0
13	MBC	0.77	0.07
14	PBC	0	0
15	PBC	0	0
16	PBC	0	0
17	MBC	0	0
18	MBC	133.48	72.70
19	NC	0	0
20	NC	0	0
21	PBC	0	0
22	MBC	0	0
23	NC	0	0
24	PBC	0	0
25	PBC	0	0
26	MBC	1.54	0.43
27	MBC	0.34	0.07
28	PBC	0	0
29	MBC	162.45	137.55
30	MBC	0	0

RGE=relative gene expression; AOD=assay on demand; MBC=metastatic breast cancer patient; PBC=primary breast cancer patient; NC= negative control patient.

. A strong and highly significant concordance was found between the two primer/probe sets (*N*=30, Spearman *ρ*=0.9985, *P*<0.0001).

### Reverse transcriptase–polymerase chain reaction

Only in 11 out of 14 specimens from the control samples, and 25 out of 29 specimens from patients with MBC, RNA of good quality was isolated. These samples were used for further analysis.

In none of the 11 negative control samples, was hMAM expression measurable by RT–PCR. On the other hand, CK-19 mRNA could be quantified in all control samples, with a median RGE of 0.57 (range 0.22–0.78) (Table 3

**Table 3:** Detection of disseminated epithelial cells by ICC and RT-PCR in bone marrow aspirates from control patients and patients with metastatic breast cancer

	**Control patients**	**MBC patients**
*ICC (#/10*^*6*^)		
*N*	14	24
Median	0	1
Minimum	0	0
Maximum	0	2280
No. of pt >cutoff	0	15/24
		
*RGE CK-19*		
*N*	11	25
Median	0.57	2.9
Minimum	0.22	0.25
Maximum	0.78	82409
No. of pt >cutoff	1	20/25
		
*RGE hMAM*		
*N*	11	25
Median	0	0
Minimum	0	0
Maximum	0	262.7
No of pt >cutoff	0	10/25

MBC=metastatic breast cancer; ICC=immunocytochemistry; RGE=relative gene expression; hMAM=human mamaglobin; RT-PCR=reverse transcriptase-polymerase chain reaction.

). Taking the 95 percentile from the CK-19 RGE (0.77) of the negative control group as cutoff, 20 of the 25 (80%) BM aspirates from the MBC patients had an increased CK-19 expression. The RGE of CK-19 of this patient group, ranged between 0.25 and 82 409 (med 2.9) and the RGE of hMAM between 0 and 262.7 (med 0). In 10 of the 25 (40%) samples, hMAM expression was measurable.

With the exception of one sample, all patients with detectable hMAM mRNA also had an increased CK-19 expression.

A statistically significant difference was observed between the RGE of CK-19 of control patients and MBC patients (Mann–Whitney: *P*=0.005). The RGE of hMAM was >0 in 10 out of 25 MBC patients (Fisher's exact test: *P*=0.016).

### Correlation RT–PCR and ICC

A strong correlation was found between CK-19 and hMAM RGE in the BM (*N*=25, Spearman *ρ*=0.7, *P*=0.003, CI=0.2–0.8). In 20 of the 29 patients with MBC, results for detection of DEC were obtained by the two techniques: ICC and PCR. A strong correlation between ICC detection of CK-positive cells and CK-19 RGE (*N*=20, Spearman *ρ*=0.9, *P*<0.0001, CI 0.8–1) or hMAM RGE (*N*=20, Spearman *ρ*=0.6, *P*=0.003, CI 0.2–0.8) was observed.

In 25% (five out of 20) of the patient samples with a CK-19 RGE above the cutoff, no cytokeratin-positive cells were detected. On the other hand, there was no sample that had a positive ICC result and an RGE of CK-19 below the cutoff (concordance: 75%, Mc Nemar: *P*=0.06 ‘fair’ *κ* value of 0.42). (Table 4

**Table 4:** Comparison between ICC and CK-19 RT-PCR for the detection of disseminated epithelial cells in bone marrow aspirates

**Number of patients**	**ICC=0**	**ICC >0**
RGE CK-19 >0.77	5	12
RGE CK-19 <0.77	3	0
		
RGE hMAM >0	1	7
RGE hMAM=0	7	5

ICC=immunocytochemistry; RT-PCR=reverse transcriptase–polymerase chain reaction; RGE=relative gene expression; hMAM=human mammaglobin.

). For hMAM expression, a concordance with ICC of 70% is found (Mc Nemar: *P*=0.22), with a fair *κ* value as well (0.42).

## DISCUSSION

Current guidelines for the adjuvant treatment of patients with lymph node negative breast cancer result in overtreatment, with inherent disadvantages and even health risks. In conjunction with increasingly effective adjuvant strategies, a more optimal patient selection is thus a matter of critical importance. Genomic expression profiling is one potential modality of obtaining a powerful predictor for patients with localised breast cancer (Van de Vijver *et al*, 2002; Van’t Veer *et al*, 2002). The presence and the number of DEC in patients with breast cancer at diagnosis may be a marker for early metastasis. Numerous studies spanning a period of more than 30 years and a recent pooled analysis have corroborated the prognostic impact of the presence of the so-called ‘BM micrometastasis’ (Braun *et al*, 2003). The studies are all based on the morphological detection of epithelial cells in BM as demonstrated by specific immunostaining.

Immunocytology is regarded as the standard method for tumour cell detection. This observer-dependent method is hampered by a low sensitivity and is labour intensive. False-positive staining of haematological cells (Borgen *et al*, 1999) can occur. With the Epimet® staining kit, we observed no doubtful cells in BM of our control population.

Molecular diagnostics have, in this respect, the advantage of being both sensitive and easy to integrate in laboratory practice, but the lack of morphological proof of a signal necessitates validation experiments. Reverse transcriptase–polymerase chain reaction assays are more suitable for routine laboratory analysis. Some RT–PCR assays have been developed to improve the sensitivity of the detection of DEC in BM (Ghossein and Rosai, 1998; Ghossein *et al*, 1999; Lambrechts *et al*, 1999; Slade *et al*, 1999; Zach *et al*, 1999; Zhong *et al*, 1999; Berois *et al*, 2000; Hu and Chow, 2000; Zippelius and Pantel, 2000; Corradini *et al*, 2001).

The optimal approach for the RT–PCR-based quantification of DEC in breast cancer would be the amplification of breast cancer cell-specific mRNA transcripts. The alternative is to exploit epithelial cell-specific gene expression. Amplification of CK-19 mRNA is used in many studies, and false positivity due to illegitimate expression by nonepithelial cells is reported (Chelly *et al*, 1989; Ko *et al*, 2000). In the present study, CK-19 mRNA transcripts were detectable in all BM samples of the control patients. Owing to this, a quantitative interpretation was adopted, introducing a cutoff threshold above which a sample was considered positive (the 95th percentile of the RGE for CK-19 of the control patients). The final aim is to select patients who do not need adjuvant chemotherapy, but with minimal risk of failing to identify those patients with very low levels of DEC. False-negative RT–PCR results thus have to be avoided. For this reason, a more stringent cutoff was not considered appropriate.

A single marker to detect and quantify DEC in BM or other samples might lead both to false-negative and false-positive analyses. Reverse transcriptase–polymerase chain reaction according to the Taqman methodology was performed to quantify DEC in leukapheresis products of breast cancer patients by the RGE using a panel of genes related to the breast gland or involved in breast cancer: maspin, mammaglobin and c-ErbB-2 (Leone *et al*, 2001). Maspin and mammaglobin were not expressed in control samples, and the mammaglobin expression was the most sensitive single marker to detect DEC. An additional reason for false negativity is the inherent mutator phenotype of cancer resulting in heterogeneous gene expression patterns, both topographically and temporally, within a tumour. Gene expression patterns of different DEC of a patient with breast cancer probably show the same heterogeneity (Klein *et al*, 2002; Schmidt-Kittler *et al*, 2003). Gene expression most probably is also context dependent, with other genes being expressed as dictated by different extracellular components in different end organs.

Mammaglobin expression was used as a second marker to quantify DEC in the BM of patients with breast cancer. The specificity of this marker for the detection of breast cancer cells in haematopoietic products has been evaluated in several studies, but, with the exception of one study (Suchy *et al*), without additional quantitative analysis (Leygue *et al*, 1999; Zach *et al*, 1999; Suchy *et al*, 2000; Corradini *et al*, 2001; Silva *et al*, 2002; Lin *et al*, 2003).

In our study population, 80% of the patients with metastatic breast cancer had an elevated CK-19 expression in their BM aspirates. Only in 40% of these patients with metastatic breast cancer was hMAM mRNA amplificable.

To check for the specificity of our home-designed primer and probe combination, we compared it with a predesigned ready to use primer and probe set prepared by Applied Biosystems (Assay-on demand Gene Expression Product number Hs00267190_m1). Primers and probe for this assay are designed using all available public and private genome databases. In order to design a robust assay for hMAM transcript detection, SNPs, repeats, sequence discrepancies and penalised regions of high homology are masked. All the components are quality controlled tested and functionally tested on human cDNA. No discrepancies were observed when RGE was measured with our home-made primers/probe set and compared with the predeveloped set (Figure 2). All samples with no hMAM expression in our designed assay also had no mammaglobin amplification with the predeveloped assay.

Also the variability in mammaglobin expression in different breast (cancer) tissues may explain the variability in expression. Leone *et al* (2001) found a high intertumoral variability of mammaglobin expression levels. Zach *et al* (1999) confirmed that different breast cancer cell lines show different mammaglobin expression levels. A similar variable expression between different cell lines was observed during our studies with no hMAM expression in MDA-MB 231 cells, weak expression in MCF-7 cells and strong expression in MDA-MB361 cells.

The group of Nunez-Villar (Nunez-Villar *et al*, 2003) suggested that breast cancer specimens with an elevated hMAM expression are better differentiated, have a lower proliferation rate and an increased hormone dependence. They observed a significant inverse correlation between a high mammaglobin expression and the most important negative clinical prognostic factor for breast cancer being axillary nodal involvement.

Suchy *et al* (2000) remarked that mammaglobin-expressing cells in BM may define a specific subclass of tumour cell phenotypes. Overexpression of mammaglobin may be restricted to a subset of tumour cells. Detection of hMAM expression in BM aspirates is restricted to cancer patients, therefore indicating absolute tumour specificity. On the other hand, absence of hMAM expression does not exclude the presence of tumour cells. Probably the presence of a positive CK-19 signal and a positive hMAM expression will be more specific for the presence of DEC. Grunewald *et al* (2000) observed that hMAM expression is a superior marker for the detection of breast cancer cells in peripheral blood as compared with cytokeratin-19. The nested RT–PCR assay was not able to measure mRNA levels quantitatively and thus resulted in false-positive results for CK-19 expression. Therefore, only hMAM mRNA expression correlated with clinical parameters. We will use both mRNA markers (CK-19 and hMAM) in larger clinical studies to evaluate this usefulness in the detection of DEC and to correlate them with clinico pathological parameters. To quantify mRNA expression in a sample, it is essential to correct for differences in the amount of total RNA added to a reaction, to compensate for different RT efficiencies, to compensate for PCR inhibitors in the sample and for possible RNA degradation occurring during sample processing. Therefore, an endogenous reference is used. Different articles describe the difficulties in choosing a suitable housekeeping gene (Kreuzer *et al*, 1999; Thellin *et al*, 1999; Bustin, 2000; Vandesompele *et al*, 2002). Two of the most used housekeeping genes for the detection of DEC in BM aspirates are GAPDH and *β*-Actin. We measured the expression of both these markers in 81 different BM samples. Since the BM aspirates show a wide variation in GAPDH expression while *β*-Actin is expressed equally between the different samples, *β*-Actin was chosen as housekeeping gene. Results of Révillion *et al* (2000) showed that GAPDH expression is associated with breast cancer cell proliferation and with the aggressiveness of tumours and therefore should not be used as a control RNA.

We used the comparative Ct method, as previously described by Livak and Schmittgen (2001), to quantify the relative gene expression with the formula 2^−ΔΔCt^. This formula is based on the assumption that the amplification efficiencies of the endogenous reference and target gene (CK19 or hMAM) are approximately equal and that the amplification efficiency is close to 1. This assumption was tested by plotting the log of input of total RNA *vs* the ΔCt value: the slope should be ⩽0.1. For CK-19 *vs* GAPDH, a slope of 0.08 was found and *vs β*-Actin 0.1. The amplification efficiencies of hMAM and GAPDH or *β*-Actin were also equal (sloop of 0.08 and 0.01). We decided that due to this validation, experiment, the comparative Ct method might be used to quantify our results relatively.

Despite the strong correlation found between quantitative ICC results and quantitative RT–PCR results, expression of RT–PCR as a number of positive cells remains difficult. Since the results are expressed relatively to a calibrator sample, it may be possible to recalculate RGE levels. For example, the maximum detectable CK-19 RGE was 82 409. This means that this sample has an 824 times higher CK-19 expression of mRNA than the calibrator sample or an expression equal to 4120 MDA-MB361 cells per million MNC.

We did not convert the RGE to number of tumour cells because we argued that the expression level of CK-19 might vary extensively between individual patients and between different tumour cells. The amount of copies in tumour cells disseminated in the BM of breast cancer patients might not only depend on the number of tumour cells but also on the extent of CK-19 or hMAM mRNA expression per single cell. It is impossible to distinguish between one cell containing 10 copies of CK-19 mRNA and 10 cells each containing one copy of CK-19 mRNA. Similarly, the expression level of CK-19/hMAM of circulating tumour cells in patients is not necessarily comparable with the expression level of the cell line used to construct a standard curve, particularly since cell lines are probably clonal with regard to target transcript copy number. It remains possible that the variation in the expression level of CK-19 mRNA in tumour cells between individual patients is overruled by the variation in the number of circulating tumour cells between these patients and that this assumption will explain the correlation found between ICC and RT–PCR results.

Yet, quantitative RT–PCR is useful for at least two reasons: (1) to establish a cutoff level to exclude false positivity and (2) long-term follow-up investigations will have to determine the prognostic information contained in the RGE.

In all, 12 out of 20 BM aspirates were positive by CK-19 RT–PCR and ICC, whereas five out of 20 samples were positive by RT–PCR but negative by ICC. Since we look for DEC in a patient population with known clinically proven metastasis, we conclude that RT–PCR is more sensitive than ICC. Similar results were found by Zhong *et al* (1999). Also Slade *et al* (1999) report a qualitative correlation between ICC and RT–PCR when these are applied to the metastatic blood samples. When analysing BM samples of primary breast cancer patients, a 50% correlation between the two techniques exists. According to them, we are working in many cases at the limits of the assays, and therefore some variation is expected because of sampling errors. In addition, the samples that were positive by RT–PCR but negative by ICC may have been so due to the superior sensitivity of PCR. Schoenfeld *et al* (1997) found some BM aspirates from breast cancer patients were RT–PCR failed to detect CK-19 mRNA where ICC stained some cytokeratin-positive cells. They suggest that these immunocytochemically detected cells were not viable or that they were dormant with low metabolic activity as defined by their inability to synthesise CK-19 mRNA. This highlights the benefit of using a second test that incorporates morphological assessment of immunostained cells when interpreting data from RT–PCR studies. Also, note that Wiedswang *et al* showed that an increased rate of detection by ICC does not improve the clinical use of DEC detection in patients with early breast cancer.

Smith *et al* found comparable results in blood samples when analysing both methodologies. They found a significant Spearman rank correlation when assessing the relationship between RT–PCR values and numbers of cells detected on ICC . From 133 specimens analysed and yielding data using both methodologies, 71% showed the same qualitative results with a moderate *κ* (=0.43).

In conclusion, while it is unproven that an increased detection rate would lead to an improved identification of patients at an increased risk of recurrence, we consider it critical that a quantitative correlation is demonstrated between ICC and RT–PCR, enabling the introduction of molecular detection techniques as part of larger clinical trials.
